# Association between meeting 24-h movement guidelines and health in children and adolescents aged 5–17 years: a systematic review and meta-analysis

**DOI:** 10.3389/fpubh.2024.1351972

**Published:** 2024-05-07

**Authors:** HanHua Zhao, Na Wu, Eero A. Haapala, Ying Gao

**Affiliations:** ^1^Department of Sports Science, College of Education, Zhejiang University, Hangzhou, China; ^2^Shanghai Innovation Center of Traditional Chinese Medicine Health Service, School of Public Health, Shanghai University of Traditional Chinese Medicine, Shanghai, China; ^3^Faculty of Sport and Health Sciences, University of Jyväskylä, Jyväskylä, Finland; ^4^Institute of Biomedicine, School of Medicine, University of Eastern Finland, Kuopio, Finland

**Keywords:** physical activity, screen time, sleep, 24-h movement guidelines, health indicators, children and adolescents

## Abstract

Twenty-four-hour movement behaviors have significant implications for physical and mental health throughout one’s lifespan. Consistent with movement behaviors, several countries have formulated and published 24-h movement guidelines. This review summarized the studies related to 24-h movement guidelines among children and adolescents from six databases (MEDLINE, EMBASE, PubMed, Web of Science, CINAHL, and SPORTDiscus). In a total of 61 studies that discussed compliance with 24-h movement guidelines, the overall adherence rate was very low (7.6%), with boys exceeding girls, children surpassing adolescents, and regional differences. A total of 39 studies examined the associations between 24-h movement guidelines and health indicators. Findings indicated that meeting all three guidelines was favorably associated with adiposity, cardiometabolic health, mental and social health, physical fitness, health-related quality of life, academic achievement, cognitive development, perceived health, dietary patterns, and myopia. Future research should utilize longitudinal and experimental designs to enhance our understanding of the associations between 24-h movement guidelines and health indicators, thereby aiding the formulation and refinement of such guidelines.

**Systematic review registration:**

https://www.crd.york.ac.uk/prospero/, CRD42023481230.

## 1 Introduction

The 24-h day encompasses physical activity (PA), sedentary behavior (SB), and sleep, collectively referred to as movement behaviors, which span a wide range of energy expenditure levels (1). Over the past decades, studies have traditionally examined the health effects of these behaviors in isolation. Higher levels of PA, lower SB, and adequate sleep are favorably associated with adiposity, motor development, and other health indicators in children and adolescents (2, 3). Conversely, lack of PA, prolonged SB, or excessive screen time (ST), and insufficient sleep have been linked to adverse health outcomes (2, 3). More recently, researchers have begun to examine the combined effects of 24-h movement behaviors on health. Studies have shown different general combinations (e.g., all three, none) and special combinations (e.g., high PA and high SB; high PA and low sleep) (4).

In 2016 and 2017, Canada and Australia developed 24-h movement guidelines for children and youth aged 5–17 years (5–7). Subsequently, the World Health Organization, the United Kingdom, New Zealand, South Africa (8–11), the Asia-Pacific region, and New Zealand (12, 13) have adopted the 24-h movement guidelines. These guidelines recommend that children and adolescents spend ≥60 min/day engaged in moderate-to-vigorous physical activity (MVPA) on ≥5 days/week, limit recreational ST to ≤2 h/day, and get between 9 and 11 h of sleep per night (aged 5–13 years) or between 8 and 10 h per night (aged 14–18 years) (5–7).

Despite countries being currently engaged in the development of 24-h movement guidelines, there remains a lack of comprehensive data and systematic reviews specifically on children and adolescents. Therefore, this review aimed to synthesize existing evidence for children and adolescents 1) to assess adherence and conduct a meta-analysis of global 24-h movement guidelines and 2) to determine the association between meeting both the general combination and the specific combination of 24-h movement guidelines with health outcomes.

## 2 Methods

### 2.1 Data source and search strategy

The review was registered with the International Prospective Register of Systematic Review (PROSPERP) (Registration ID CRD42023481230) and conducted using the Preferred Reporting Items for Systematic Review and Meta-Analysis (PRISMA) (14). Two researchers (HHZ and NW) searched six databases (MEDLINE, EMBASE, PubMed, Web of Science, CINAHL, and SPORTDiscus) using the following search strategy: ((24 h*)OR(movement behavio*)OR(movement guidelin*)OR(physical activit* AND sedentary behavio* AND slee*)). The research terms were customized for each research database. Detailed search terms and procedures can be found in Supplementary Table S1.

### 2.2 Inclusion criteria

The eligibility criteria included: (1) data restricted to the period from 2016 to 2022, as this timeframe encompasses the release of influential 24-h movement guidelines in 2016 (5), (2) study design: cross-sectional and longitudinal study, with separate analysis, (3) participants: aged 5–18 years children (aged 5–12 years) and adolescent (aged 13–18 years), (4) exposure: including two or more behaviors from 24-h movement guidelines, specifically MVPA, ST, and sleep, assessed by either wearable devices or self-report measurements, and (5) health outcome: at least 1 health indicator, including adiposity, cardiometabolic health, physical fitness, mental and social health, health-related quality of life, academic achievement, cognitive development, dietary patterns, and myopia.

### 2.3 Study selection and data extraction

**Figure 1:**
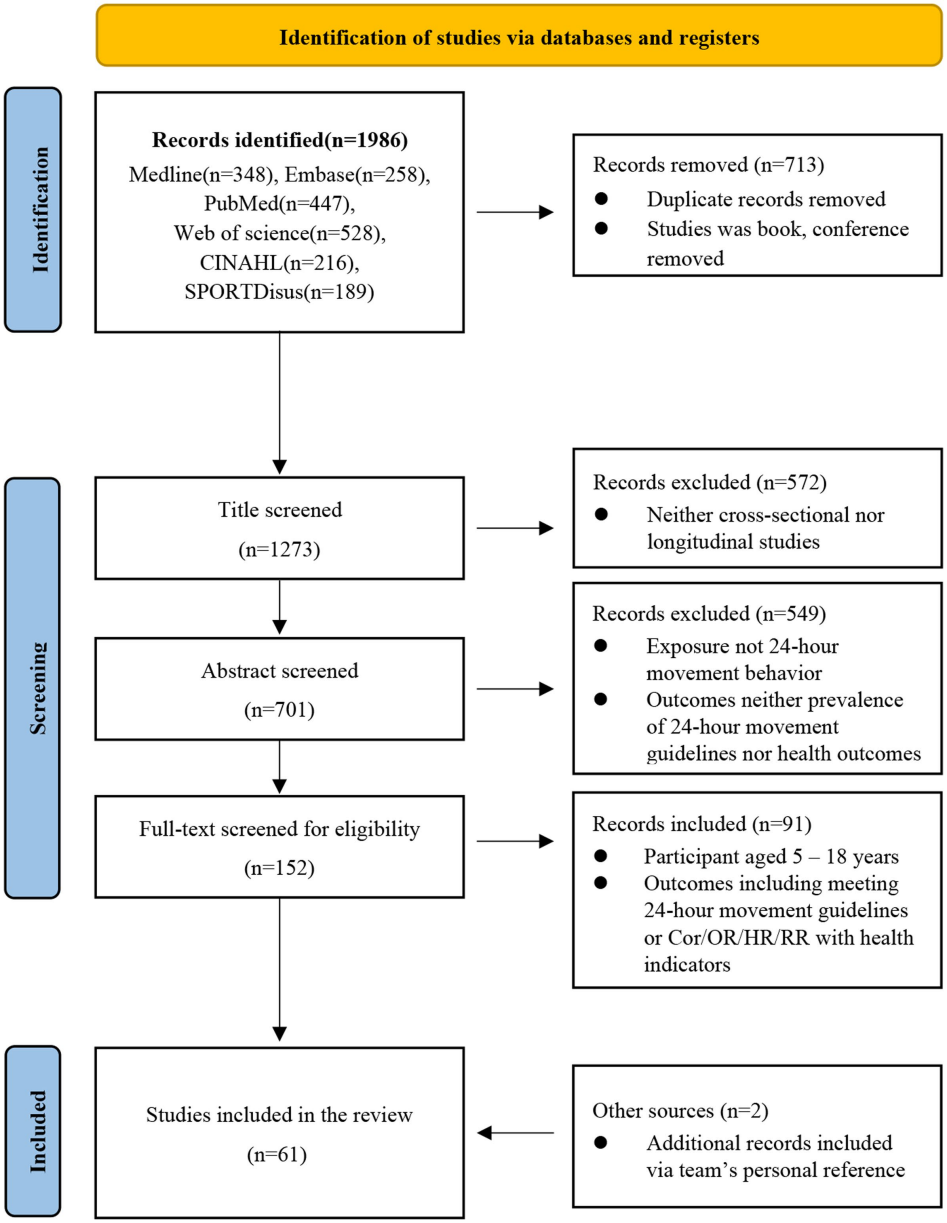
The flow chart of research results.

After the identification of studies through database searching, all acquired articles were stored in an EndNote X9 reference manager (Thompson ISI Research Soft, Philadelphia, PA, United States). Initially, duplicate records were removed with automated deduplication inside the software. Following this, two researchers (HHZ and NW) screened title, abstract, and full-text articles independently for potentially relevant records. Two researchers (HHZ and NW) examined all full-text articles to determine eligibility. Disagreements between the two researchers were resolved by discussion or with a third researcher (YG). In addition to searching the database, the review team checked their personal reference lists and Google Scholar for potential studies. A flowchart regarding the procedure can be found in Figure 1.

All included articles were summarized in table format using Microsoft Excel and described the following study characteristics: author, publication year, country, study design, population, sample size (Table 1), exposure, exposure measures, outcomes, and results (Supplementary Tables S2, S3).

**Table 1:** Association between 24-h movement guidelines and health-related indicators.

Author, year	Study design and sample	PA	Sleep	ST	Health-related indicators	General combination	Specific combination
Laruson, 2016	Cross-sectional, United States, grades 9–12, *N* = 9,589	Self-reported	Self-reported	Self-reported	Adiposity	✓	✓
Roman-vainas, 2016	Cross-sectional, 12 countries, aged 9–11, *N* = 6,129	4–7d by ACC	3–7d by ACC	Self-reported	Adiposity	✓	✓
Carson, 2017	Cross-sectional, Canada, aged 6–17, *N* = 4,157	4–7d by ACC	Self- or parent-reported	Self- or parent-reported	Adiposity, cardiometabolic, physical fitness, mental health	✓	✓
Jassen, 2017	Cross-sectional, Canada, aged 10–17, *N* = 17,000	Self-reported	Self-reported	Self-reported	Adiposity, mental and social health	✓	✓
Katzmaryzk, 2017	Cross-sectional, United States, aged 5–18, *N* = 357	Self- or parent-reported	Self- or parent-reported	Self- or parent-reported	Adiposity, cardiometabolic	✓	✓
Sampasa, 2017	Cross-sectional, 12 countries, aged 9–11, *N* = 6,106	4–7d by ACC	3–7d by ACC	Self-reported	Health-related quality of life	✗	✓
Lee, 2018	Cross-sectional, Korea, aged 12–17, *N* = 50,987	Self-reported	Self-reported	Self-reported	Psychological	✓	✓
Walsh, 2018	Cross-sectional, United States, aged 9–10, *N* = 4,524	Self-reported	Self-reported	Self- or parent-reported	Global cognitions	✓	✓
Guerrero, 2019	Cross-sectional, Canada, aged 8–11, *N* = 4,524	Self-reported	Self-reported	Self- or parent-reported	Mental health	✗	✓
Thivel, 2019	Cross-sectional,12 countries, aged 9–11, *N* = 5,973	4–7d by ACC	3–7d by ACC	Self-reported	Dietary patterns	✗	✓
Zhu, 2019	Cross-sectional, United States, aged 6–17, *N* = 35,688	Self- or parent-reported	Self- or parent-reported	Self- or parent-reported	Mental health	✓	✓
Zhu, 2020	Cross-sectional, United States, aged 10–17, *N* = 30,478	Parent-reported	Parent-reported	Parent-reported	Body weight	✓	✓
Chemtob, 2021	Longitudinal, Canada, aged 8–10, N = 630, aged 10–12, *N* = 564, aged 15–17, *N* = 377	4–7d by ACC	Self-reported	Self-reported	Adiposity	✓	✗
Hui, 2020	Cross-sectional, 8 cities, aged 13.63 ± 1.01, *N* = 12,590	Self-reported	Self-reported	Self-reported	Body fat percentage	✓	✓
Sampasa, 2021	Cross-sectional, Canada, aged 11–20, *N* = 10,236	Self-reported	Self-reported	Self-reported	Substance use	✓	✓
Sampasa, 2021	Cross-sectional, United States, aged 9–11, *N* = 11,875	Self-reported	Self-reported	Parent-reported	Mental health	✓	✓
Tanaka, 2020	Cross-sectional, Japanese, aged −12, *N* = 243	3–7d by ACC	Self- and parent-reported	Self- and parent-reported	Physical fitness	✓	✓
Watson, 2022	Cross-sectional, Australia, aged 11–12, *N* = 1,279	Self-reported or 4–8d by ACC	Self-reported or 4–8d by ACC	Self-reported	Academic achievement	✓	✓
Tanaka, 2021	Cross-sectional, Japanese, aged 6–12, *N* = 902	3–7d by ACC	Self-reported	Self-reported	Adiposity	✓	✓
Chen, 2021	Cross-sectional, China, grades 4–12, *N* = 114,072	Self-reported	Self-reported	Self-reported	Adiposity	✓	✓
Burns, 2020	Cross-sectional, United States, grades 9–12, *N* = 1849	Self-reported	Self-reported	Self-reported	Mental health	✓	✓
Khan, 2021	Cross-sectional, Australia, aged 12–13, N = 3,096	Self-reported	Self-reported	Parent-reported	Quality of life	✓	✓
Shi, 2020	Cross-sectional, Hong Kong, aged 11–18, *N* = 1,039	ACC	ACC	Self-reported	Adiposity	✓	✓
Howie, 2020	Cross-sectional, Australia, grades 5–12, *N* = 934	Self-reported	Self-reported	Self-reported	Academic achievement	✓	✓
Guimaraes, 2020	Cross-sectional, Canada, aged 12–17, *N* = 276	Self-reported	Self-reported	Self-reported	Adiposity, quality of life, mental health	✓	✓
Sampasa, 2020	Cross-sectional, Canada, grades 7–12, *N* = 10,183	Self-reported	Self-reported	Self-reported	Mental health	✓	✓
Samapsa, 2022	Cross-sectional, Spanish, aged 11–16, N = 1,290	Self-reported	Self-reported	Self-reported	Academic achievement	✓	✓
Chen, 2022	Cross-sectional, China, aged 11–17, *N* = 3,870	Self-reported	Self-reported	Self-reported	Physical fitness	✓	✓
Tapia-serrano, 2022	Cross-sectional, Spanish, aged 11–16, *N* = 1,276	Self-reported	Self-reported	Self-reported	Physical fitness	✗	✓
Zeng, 2022	Cross-sectional, China, aged 7–12, *N* = 376	4–7d by ACC	Self-reported	Self-reported	Executive function	✓	✓
Jakubec, 2020	Cross-sectional, Czech, aged 8–18, *N* = 679	ACC	Acc	Parent- or self-reported	Adiposity	✓	✓
Yang, 2022	Cross-sectional, China, aged 6–18, *N* = 34,887	Parent- or self-reported	Parent- or self-reported	Parent- or self-reported	Adiposity, cardiometabolic	✓	✓
Cai, 2023	Cross-sectional, China, aged 13–15, *N* = 48,698, aged 16–18, *N* = 47,147	Self-reported	Self-reported	Self-reported	Physical fitness	✓	✓
Swindell, 2022	Cross-sectional, Kenya, aged 11.1 ± 0.8, *N* = 539	ACC	Self-reported	Acc	–	✗	✗
Lu, 2021	Cross-sectional, China, grades 4 and 5, *N* = 5,537	Self-reported	Self-reported	Self-reported	Depressive and anxiety	✓	✓
Kyan, 2022	Cross-sectional, Japan, aged 10–11, *N* = 2,408, aged 13–14, *N* = 4,360	Self-reported	Self-reported	Self-reported	Self-rated health	✗	✓
Costa, 2021	Cross-sectional, Brazil, aged 14–18, *N* = 867	Self-reported	Self-reported	Self-reported	–	✗	✗
Lu, 2023	Cross-sectional, China, aged 14–17, *N* = 6,032	self-reported	Self-reported	Self-reported	Cognitive difficulties	✗	✓
Zhao, 2023	Cross-sectional, China, aged 5–13, *N* = 1,423	Parent-reported	Parent-reported	Parent-reported	Myopia	✓	✓
Leppanen, 2021	Cross-sectional and Longitudinal, Finland, aged 6–8, *N* = 485	Heart rate and Actiheart	Parent- or self-reported	Heart rate and Actiheart	Cardiometabolic risk	✓	✓
Sun, 2023	Cross-sectional, China, aged 11.6 ± 0.8, *N* = 1,098	Self-reported	Self-reported	Self-reported	Subjective wellbeing	✓	✓
Hansen, 2022	Cross-sectional, German, aged 9–12, *N* = 6,451, aged 13–18, *N* = 9,335	Self-reported	Self-reported	Self-reported	–	✗	✗
Lien, 2020	Cross-sectional, Canada, grades 7–12, *N* = 10,160	Self-reported	Self-reported	Self-reported	Academic achievement	✗	✓
Guedes, 2022	Cross-sectional, Brazil, aged 14–18, *N* = 306	Self-reported	Self-reported	Self-reported	Cardiometabolic health markers	✓	✓
Bang, 2020	Cross-sectional, Canada, aged 5–11, *N* = 2,773, aged 12–17, *N* = 1,477	ACC	Parent- or self-reported	Parent- or self-reported	Psychosocial health	✓	✓
Fung, 2023	Cross-sectional and longitudinal, United States, aged 9–14, *N* = 10,574	Parent- or self-reported	Parent- or self-reported	Parent- or self-reported	Cognitive development	✗	✓
Suchert, 2023	Cross-sectional, German, aged 9–17, *N* = 17,433	Self-reported	Self-reported	Self-reported	–	✗	✗
Sampasa, 2022	Cross-sectional, Canada, grades 7–9, *N* = 5739/6960	Self-reported	Self-reported	Self-reported	Self-rated physical and mental health	✓	✓
Zhou, 2022	Cross-sectional, China, grades 1–6, *N* = 978	Self-reported	Self-reported	Self-reported	Body composition	✓	✓
Ma, 2022	Cross-sectional, China, grades 4–5, *N* = 2,405	Self-reported	Self-reported	Self-reported	Internet addiction	✓	✓
Zhang, 2022	Cross-sectional and longitudinal, China, grades 7–12, *N* = 6,984	Self-reported	Self-reported	Self-reported	Mental health problem	✓	✗
Garcia, 2023	Cross-sectional, United States, grades 7–12, *N* = 6,984	Self-reported	Self-reported	Self-reported	Obesity	✗	✓

ACC, accelerometer.

### 2.4 Data synthesis

Stata was planned for the meta-analyses (Version 17, StataCorp., College Station, TX, United States) using the “metan” command and Metaprop tests. Metaprop was implemented to perform meta-analyses pooling adherence to 24-h movement guidelines in Stata. Heterogeneity was calculated using the I-square (I^2^) values. If I^2^ > 50%, the random-effects model was selected, and if I^2^ < 50%, the fixed-effects model was chosen. Sub-group analyses were conducted based on age groups, gender, and geographical region. The “metan” command was used to pool effects [odds ratios (ORs)] between 24-h movement guidelines and health indicators in Stata. Random-effects models were used in the data analysis to combine effects and calculate 95% confidence intervals (95% CI) when a sufficient number of studies reported associations for the same outcomes.

## 3 Result

### 3.1 Description of studies

A total of 61 studies (15–75) assessed adherence to 24-h movement guidelines (Supplementary Table S4). Out of these, 44 (16–25, 27–34, 36, 37, 40, 42, 43, 46–59, 61–65, 67, 69–74) reported the proportions of participants meeting none of the guidelines, 30 studies (17–25, 28–31, 36, 40, 42, 43, 46, 49–51, 53, 55, 61, 63, 65, 69, 71, 72, 74) reported meeting only one guideline, 30 studies (17–22, 24, 25, 28–31, 36, 40, 42, 43, 45, 46, 49–51, 53, 55, 61, 63, 65, 69, 71, 72, 74) reported meeting two guidelines, and 60 studies (16–75) reported meeting all three guidelines. For the specific combination of 24-h movement guidelines, 61 studies (15–75) reported meeting the MVPA guidelines only, 60 studies (15–35, 37–75) reported meeting the ST guidelines only, 61 studies (15–75) reported meeting the sleep guidelines only, and 45 studies (16, 17, 19, 20, 22–29, 31–37, 39, 42, 43, 47, 48, 50–61, 65–67, 70–75) reported meeting any two combinations.

A total of 39 studies (16–75) reported overall adherence to 24-h movement guidelines. Regarding age group, 26 studies (16, 20, 21, 23–25, 27, 28, 31, 33–37, 48, 54, 56, 59–62, 66, 67, 70, 71, 74) focused on children and 22 studies (20, 22, 26, 28, 30–32, 38, 46, 48, 52, 53, 56–58, 62, 64, 66, 69, 72–74) focused on adolescents. In terms of gender, 19 studies (15, 20, 29, 30, 32, 33, 36, 37, 41, 42, 45, 47, 51, 53, 55, 62, 63, 65, 75) included both boys and girls. In terms of geographical region, 18 different countries were identified, including 3 in Africa (16, 25, 54), 20 in Asia (16, 22, 30, 34, 37, 39, 41, 44, 51–53, 55, 56, 58, 59, 62, 70–72, 75), 10 in Europe (16, 26, 42, 48, 50, 60, 62, 63, 68, 70), 4 in Oceania (16, 35, 43, 45), and 22 in North America (16–20, 23, 24, 28, 29, 31–33, 38, 40, 46, 47, 49, 64, 66, 67, 69, 73).

A total of 47 cross-sectional studies (15–19, 21–24, 27–29, 31–35, 37, 40, 41, 43–47, 50–53, 55, 56, 59–61, 64–67, 69–77) examined the association between 24-h movement guidelines and health indicators (Supplementary Table S2). The health outcomes described were adiposity (*n* = 17) (15–19, 29, 31, 37, 41, 44, 46, 52, 60, 70, 73–75), cardiometabolic health (*n* = 5) (17, 19, 60, 65, 74), physical fitness (*n* = 6) (17, 34, 53, 69, 76, 77), mental and social health (*n* = 17) (17, 18, 22, 24, 28, 32, 33, 40, 46, 47, 55, 61, 66, 67, 69, 71, 72), health-related quality of life (*n* = 4) (21, 43, 46), academic achievement (*n* = 4) (35, 45, 50, 64), cognitive development (*n* = 4) (23, 51, 58, 67), dietary patterns (*n* = 1) (27), perceived health (*n* = 2) (46, 56), and myopia (n = 1) (59).

A total of four longitudinal studies (31, 60, 67, 72) examined the association between 24-h movement guidelines and health indicators (Supplementary Table S3), including adiposity (*n* = 3) (31, 60, 67), cardiometabolic health (*n* = 1) (60), and mental and social health (*n* = 2) (67, 72).

### 3.2 Compliance with 24-h movement guidelines

**Figure 2:**
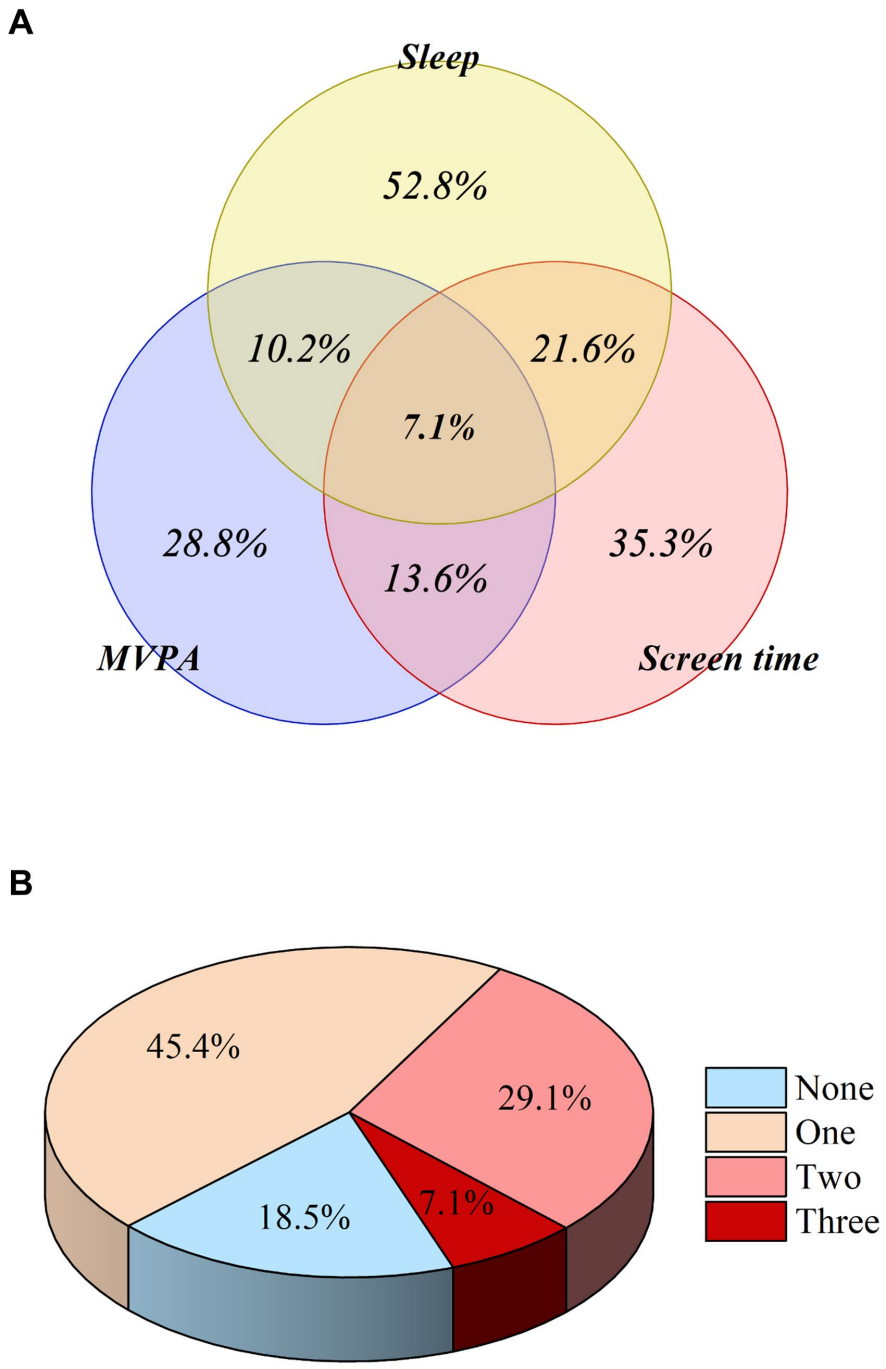
Proportion of children and adolescents meeting the specific combination **(A)** and general combination **(B)** of 24-h movement guidelines.

Figure 2 shows the proportions of children and adolescents meeting the special combination and the general combination of 24-h movement guidelines. 28.8 ± 3.8, 35.3 ± 4.0, 52.8 ± 3.8% meeting the MVPA, ST, sleep guidelines and 18.5 ± 2.1, 45.4 ± 2.5, 29.1 ± 2.0, 7.1 ± 1.0% meeting None, One, Two, Three guidelines, respectively.

**Figure 3:**
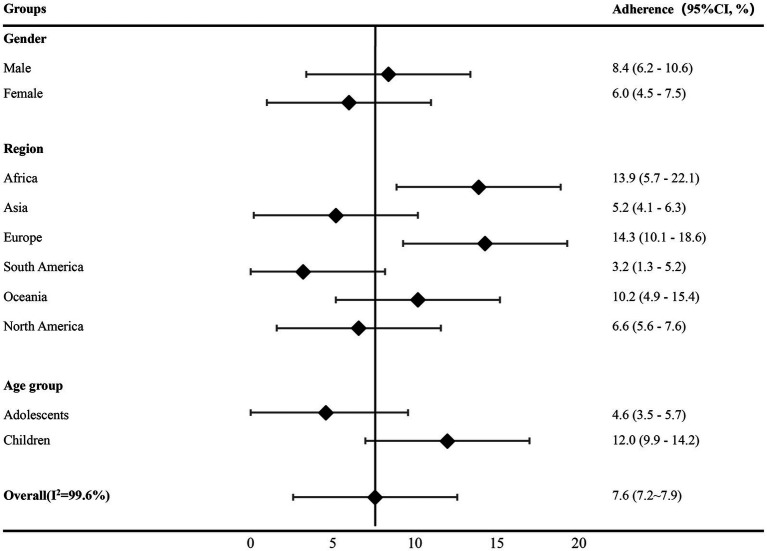
Forest plot of the overall adherence to 24-h movement guidelines by gender group, geographical region, and age group; 95% CI, 95% confidence interval.

Figure 3 illustrates overall adherence to 24-h movement guidelines across age groups, geographical regions, and gender. Adherence to guidelines was higher in male subjects than in female subjects and in children than in adolescents. Regarding geographical regions, we noted variations in overall adherence, with lower rates observed in South America (3.2%) (95% CI:1.3–5.2%) and higher in Europe (14.3%) (95% CI,10.1–18.6%).

### 3.3 Association between meeting 24-h movement guidelines and health indicators

#### 3.3.1 Adiposity

In this review, adiposity indicators included body mass index (BMI)/ body mass index z-score (BMIz), waist circumference (WC), body fat (BF/BF%/FM/FM%), and waist-to-height ratio (WHtR). The investigation into the associations between adherence to 24-h movement guidelines and BMI/BMIz was conducted in 15 cross-sectional studies (15–19, 29, 31, 37, 41, 44, 46, 60, 73–75), including 14 studies adhering to the general combination and 14 studies adhering to the specific combination. Of these studies, 10 of 14 reported that meeting the general combination was associated with a lower risk of BMI or BMIz or being obese or overweight. By conducting meta-analyses on five studies (15, 17, 29, 31, 41) with consistent exposures and outcomes, the pooled analysis revealed a significant association. Individuals not meeting any guidelines, compared to meeting all three guidelines, exhibited increased odds of BMI/BMIz (OR = 1.39, 95% CI 1.3–1.49, I^2^ = 93.9% *p* = 0.000) (see Supplementary Figure S1). Furthermore, 10 of 14 studies reported that meeting specific combinations was associated with lower BMI or BMIz or lower risk of being obese or overweight. The majority of studies (8/10) support adhering to MVPA guidelines or combining with MVPA to result in lower levels of adiposity/BMI, etc.

The associations between adherence to 24-h movement guidelines and WC were examined in five cross-sectional studies (17, 19, 31, 60, 73), including four studies that adhered to the general combination and four studies that adhered to the specific combination. Out of these, three of four studies reported that adherence to general combinations was associated with lower WC, whereas the other studies (31) reported meeting none of the guidelines was not associated with WC among children aged 8–10 years and early adolescents aged 10–12 years. Among the specific combinations, one study (17) found that meeting specific combinations was not significantly associated with WC. In contrast, a separate study demonstrated a significant association between meeting MVPA or ST and sleep guidelines and lower WC.

The associations between adherence to 24-h movement guidelines and BF were examined in five cross-sectional studies (19, 31, 52, 70, 74). Three of five studies conducted on meeting general combinations presented an association with lower BF, and two of five studies showed no association. In addition, three of five studies reported insignificant associations between meeting the specific combinations and BF, with the MVPA, ST, or combination specifically showing lower risk.

The associations between adherence to 24-h movement guidelines and WHtR were examined in one cross-sectional study (31). Meeting none of the guidelines was cross-sectionally associated with a higher WHtR among children aged 8–10 years, but no association was observed among early adolescents aged 10–12 years.

Three longitudinal studies (31, 60, 67) investigated the associations between adherence to 24-h movement guidelines and adiposity, including BMI/zBMI, BF%, WC, and WHtR. All studies conducted on meeting all three guidelines at baseline were inversely associated with adiposity at 2-year follow-up, with specific combinations emphasizing the MVPA and sleep guidelines.

#### 3.3.2 Cardiometabolic health

The associations between adherence to 24-h movement guidelines and cardiometabolic biomarkers were investigated in four cross-sectional studies (17, 19, 65, 74). Cardiometabolic biomarkers included systolic blood pressure (SBP), diastolic blood pressure (DBP), subcutaneous adipose tissue (SAT), visceral adipose tissue (VAT), triglycerides, HDL cholesterol, C-reactive protein (CRP), insulin, and glucose. Findings from the general combinations of 24-h movement guidelines indicated that meeting none of the guidelines was associated with higher SBP, higher triglycerides, lower HDL cholesterol, higher CRP, and higher Insulin (17), and meeting all three guidelines was associated with lower SAT, lower VAT, lower triglycerides, and lower glucose (19). Regarding adherence to the specific combinations, all four studies suggested that meeting either the individual or combined guidelines did not show significant associations with most cardiometabolic biomarkers, such as BP, SAT, VAT, triglycerides, HDL cholesterol, insulin, and glucose levels (17, 19).

One longitudinal study (60) explored the associations between adherence to 24-h movement guidelines and cardiometabolic health. The study revealed that meeting all three guidelines at baseline was inversely associated with insulin and CRS, with no association found for glucose, triglycerides, HDL cholesterol, SBP, or DBP at 2-year follow-up. The specific combination emphasized MVPA and MVPA and ST guidelines.

#### 3.3.3 Physical fitness

Six cross-sectional studies (17, 34, 53, 69, 76, 77) examined the associations between adherence to 24-h movement guidelines and physical fitness. Among the general combination and physical fitness, it was found that meeting all six was associated with a higher level of general fitness, cardiorespiratory fitness, muscular strength, speed, and agility; it was not associated with grip strength, sit-up, sit-and-reach, 20-m shuttle run, or flexibility (34). In terms of specific combinations, five of six studies emphasized the importance of MVPA.

#### 3.3.4 Mental and social health

The associations between adherence to 24-h movement guidelines and mental and social health were examined in 15 cross-sectional studies (17, 18, 22, 28, 32, 40, 46, 47, 55, 61, 66, 67, 69, 71, 72). Of those studies, 13 investigated adherence to the general combinations and 14 investigated specific combinations. Eight of thirteen studies showed that meeting all three guidelines was associated with lower emotional problems, not feeling stressed, fewer internalizing and externalizing behaviors, decreased loneliness and sadness, higher perceived self-efficacy, fewer suicidal ideation and suicide attempts, higher positive psychosocial health, higher prosocial behavior, higher satisfaction, lower depressive symptoms, and anxiety. Additionally, five studies indicated that non-compliance with any of the guidelines was associated with higher scores in strengths and difficulties, increased prosocial behavior, lower life satisfaction, little happiness, higher risk of internet addiction, elevated levels of anxiety, and depression. Among the specific combinations, all 14 of 14 studies reported that meeting specific combinations was associated with better mental and social health, with the importance of meeting MVPA only, ST only, sleep only, or ST and sleep.

The associations between adherence to 24-h movement guidelines and mental and social health exhibited differences based on gender and age groups. In Zhu et al. (28) study, it was reported that meeting none of the guidelines was associated with significantly increased odds of anxiety and depression in adolescents aged 12–17, but not in children aged 6–12. Sampasa’s study (47) found that meeting all three guidelines was associated with lower suicidal ideation among those aged 15–20 years, but not younger aged 11–14 years. Furthermore, the study demonstrated that meeting all three guidelines had a statistically significant association with suicidal ideation and attempts among boys, while no significant association was observed among girls.

Two longitudinal studies (67, 72) explored the associations between adherence to 24-h movement guidelines and mental and social health. One study (72) revealed adolescents who met all three guidelines at baseline displayed lower anxiety and depression symptoms at 6 months. Another study (67) emphasized that MVPA and sleep at baseline was inversely associated with cognition, psychosocial, and gray matter volumes at 2-year follow-up.

#### 3.3.5 Health-related quality of life

Three cross-sectional studies (21, 43, 46) examined the associations between adherence to 24-h movement guidelines and health-related quality of life, including two studies that met the general combination and three studies that met the specific combination. Both studies reported that adherence to meeting all three guidelines had better HRQoL. Regarding specific combinations, meeting any individual guideline or any combination was associated with higher scores in HRQoL outcomes, as indicated by three studies.

#### 3.3.6 Academic achievement

Four cross-sectional studies (35, 45, 50, 64) examined the associations between adherence to 24-h movement guidelines and academic achievement, with three studies meeting the general combination and four studies meeting the specific combination. As for the general combination, two studies (35, 50) reported that meeting all three guidelines was associated with higher academic achievement, and one study (45) showed that meeting at least two out of the three guidelines was associated with better academic achievement. Regarding specific combinations, one study (35) highlighted the significance of meeting MVPA guidelines for numeracy achievement, meeting the ST and sleep guidelines demonstrated the strongest positive association with literacy achievement. Another study (45) found that meeting the ST guidelines was associated with higher average academic index and English scores. Additionally, one study (50) that reported meeting the MVPA and sleep guidelines, both independently or together, was associated with higher academic achievement. Another study (64) showed that students who met ST or sleep displayed better academic achievement.

#### 3.3.7 Cognitive development

Four cross-sectional studies (23, 51, 58, 67) examined the associations between adherence to 24-h movement guidelines and cognitive development. Of this, two studies evaluated the general combination and four studies explored the specific combination. Two of the studies found that meeting the general combination was associated with superior cognitive development. Among the specific combinations, studies emphasized the importance of MVPA and ST and ST and sleep guidelines to promote better cognition.

#### 3.3.8 Perceived health

Two cross-sectional studies (46, 56) examined the associations between adherence to 24-h movement guidelines and perceived health. It was found that meeting all three guidelines did not show any association with perceived health when compared to meeting none. However, meeting the MVPA or MVPA and sleep or ST and sleep was associated with better-perceived health.

#### 3.3.9 Dietary patterns

The associations between adherence to the 24-h movement guidelines and dietary patterns were examined in one cross-sectional study (27). The findings revealed that meeting a higher number of guidelines was linked to improved dietary patterns, while meeting ST guidelines showed a particularly strong association with desirable dietary patterns.

#### 3.3.10 Myopia

One cross-sectional study (59) examined the associations between adherence to 24-h movement guidelines and myopia, revealing that meeting the general combination was negatively associated with a reduced risk of myopia. Among the specific combinations, encouraging sleep or ST and sleep was recommended to reduce the risk of myopia.

## 4 Discussion

We found that only 7.6% of children and adolescents met all three guidelines of the 24-h movement guidelines. This meta-analysis identified several correlates that could account for the low adherence. First, a significant association was observed between gender and meeting all three guidelines, with a higher proportion of male subjects compared to female subjects. Second, the association between the age group and meeting all three guidelines indicated that a higher proportion of children compared to adolescents fulfilled the guidelines. Third, the adherence to all three guidelines varied by region, with children from South Africa having a lower proportion.

Our study revealed low overall adherence to 24-h movement guidelines among children and adolescents. These findings are consistent with previous meta-analyses conducted in children and adolescents, reporting adherence to the guidelines ranging from 7.1 to 13% (78, 79). Compared with previous studies, the current study included similar criteria, which combined results from subjective (e.g., self-report or proxy-reported questionnaire and diary) and objective (e.g., accelerometer) measurements. Previous studies have revealed a strong correlation between different instruments that measured MVPA/sleep (80, 81). For the proportion of subjects meeting 24-h movement guidelines, adherence to all three guidelines was 3% with self-reported and 0.2% with accelerometer data (57). It seems that self-reported results may overestimate the prevalence. Further studies should separate self-reported and accelerometer measures for the 24-h movement guidelines.

Larger and more consistent evidence across studies revealed the health implications of adhering to the general combination of 24-h movement guidelines among children and adolescents. Significantly, our findings highlight the favorable health indicators associated with meeting all three guidelines as well as the unfavorable indicators associated with meeting none. Nevertheless, the evidence regarding the associations between meeting 24-h movement guidelines and improved adiposity, cardiometabolic biomarkers, and mental and social health was inconclusive across studies. For example, Shi (44), Jakubec (74), and Guimaraes (46) showed that meeting all three guidelines was not associated with BMIz (44, 46, 74) and FM% (74). It is possible that the results could be explained by almost all participants being adolescents in three studies and the lower proportion meeting all three guidelines. In the context of physical fitness, the majority of studies (34, 53, 69, 77) emphasized the importance of PA. It is known that engaging in regular PA, such as endurance activity, can improve cardiac output, oxygen-carrying capacity, and stroke volume (82). This improvement is a direct result of enhanced cardiorespiratory fitness. Resistance training potently causes increased muscle hypertrophy through an increase in myofibril size and the number of fast- and slow-twitch fibers (83), leading to improved muscular fitness.

Moreover, the neuromuscular performance (84) and reaction time (85) induced by regular PA play a central role in determining agility. Mental and social health in children and adolescents are crucial aspects. Our study observed differences in the association between 24-h movement guidelines and mental and social health based on gender and age. Reasons may vary from environment to social. One reason is that preferences for certain types of PA vary between gender groups. For example, boys might be more inclined toward team sports, while girls may prefer activities with creative components (86). These preferences can influence the association. Another reason is the increase in academic pressure (87), which is more pronounced during adolescence and impacts the association.

Meeting a general combination or specific combination may have positive implications for health indicators. Considering that children and adolescents spend most of their time in school or at home, these settings present ideal opportunities to promote holistic adherence to the 24-h movement guidelines for fostering a healthy lifestyle. Future interventions should consider targeting structured PA in the school setting. Utilizing physical education classes and recess provides opportunities for students to engage in exercise, especially given the strong association of MVPA with adiposity (15, 16, 29, 39, 44, 46, 75), and physical fitness (17, 34, 51). Moreover, home is another setting that should be considered. The modeling of parents plays an important role in promoting sufficient sleep time and limiting ST. This involves creating a conducive sleep environment, establishing consistent bedtime routines, and managing excessive screen-based device use. Encouraging ST and sleep also contributes to better-perceived health, substance use, and executive function (32, 45, 51). Significantly, a positive correlation was observed between meeting a higher number of guidelines and experiencing more favorable health indicators. Consequently, the partnership between the school and home environments holds substantial potential for generating a synergistic influence on the health outcomes of children and adolescents.

Our results need to be interpreted in light of several limitations. The major limitation of our findings was the insufficient evidence to conclude a consistent association between 24-h movement guidelines and exposure variables (e.g., academic achievement). Several plausible explanations may account for the observed associations. To address this gap, more research is needed to investigate and confirm this association. Another constraint is that most of the studies included in this analysis utilized a cross-sectional design, which precludes establishing causal associations. To address this limitation, future research should incorporate longitudinal and experimental designs to provide more robust evidence.

## 5 Conclusion

The outcomes of this review contribute to the existing body of evidence regarding adherence to the 24-h movement guidelines and their associations with health indicators in children and adolescents. The overall adherence rate is alarmingly low (7.6%), exhibiting notable disparities across age groups, genders, and geographical regions. Meeting 24-h movement guidelines was associated with favorable adiposity, cardiometabolic health, mental and social health, physical fitness, health-related quality of life, academic achievement, cognitive development, perceived health, dietary patterns, and myopia. Presently, the available evidence on health indicators is limited and inconclusive. Given that most of the included studies utilized a cross-sectional design, further research incorporating longitudinal and experimental designs is warranted to enhance comprehension of the association between 24-h movement guidelines and health indicators, as well as facilitate the development of comprehensive 24-h movement guidelines.

## Data availability statement

The original contributions presented in the study are included in the article/Supplementary material, further inquiries can be directed to the corresponding author.

## Author contributions

HHZ: Writing – original draft, Data curation, Investigation, Methodology. NW: Data curation, Investigation, Methodology, Writing – review & editing. EH: Writing – review & editing, Conceptualization, Project administration. YG: Writing – review & editing, Conceptualization, Supervision, Funding acquisition, Investigation, Project administration, Data curation.
