# Corrigendum: Association between meeting 24-h movement guidelines and health in children and adolescents aged 5–17 years: a systematic review and meta-analysis

**DOI:** 10.3389/fpubh.2024.1435964

**Published:** 2024-07-19

**Authors:** HanHua Zhao, Na Wu, Eero A. Haapala, Ying Gao

**Affiliations:** ^1^Department of Sports Science, College of Education, Zhejiang University, Hangzhou, China; ^2^Shanghai Innovation Center of Traditional Chinese Medicine Health Service, School of Public Health, Shanghai University of Traditional Chinese Medicine, Shanghai, China; ^3^Faculty of Sport and Health Sciences, University of Jyväskylä, Jyväskylä, Finland; ^4^Institute of Biomedicine, School of Medicine, University of Eastern Finland, Kuopio, Finland

**Keywords:** physical activity, screen time, sleep, 24-h movement guidelines, health indicators, children and adolescents

In the published article, there was an error in Figure 2 as published. The numbers were in the wrong place. The corrected Figure 2 appears below.

**Figure 2:**
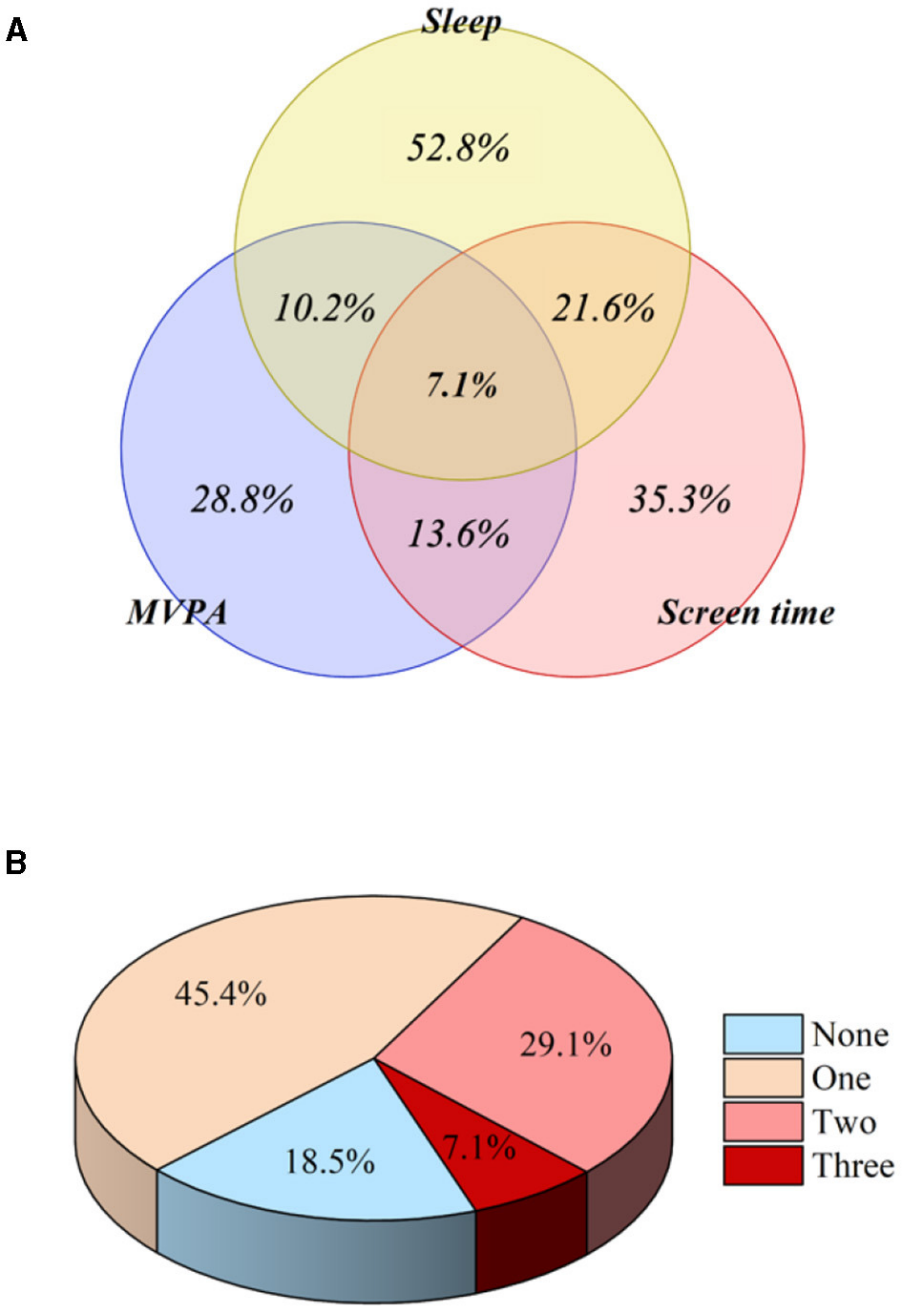
Proportion of children and adolescents meeting the specific combination **(A)** and general combination **(B)** of 24-h movement guidelines.

In the published article, there was an error. [Adherence to all three 24-h movement guidelines was updated with 7.6%].

A correction has been made to **Abstract**, Paragraph one. This sentence previously stated:

“In a total of 61 studies that discussed compliance with 24-h movement guidelines, the overall adherence rate was very low (7.1%).”

The corrected sentence appears below:

“In a total of 61 studies that discussed compliance with 24-h movement guidelines, the overall adherence rate was very low (7.6%).”

A correction has been made to **Discussion**, Paragraph one. This sentence previously stated:

“We found that only 7.1% of children and adolescents met all three guidelines of the 24-h movement guidelines.”

The corrected sentence appears below:

“We found that only 7.6% of children and adolescents met all three guidelines of the 24-h movement guidelines.”

A correction has been made to **Conclusion**, Paragraph one. This sentence previously stated:

“Overall, the overall adherence rate is alarmingly low (7.1%).”

The corrected sentence appears below:

“The overall adherence rate is alarmingly low (7.6%).”

The authors apologize for these errors and state that this does not change the scientific conclusions of the article in any way. The original article has been updated.

