# Supplementary material for: Association between meeting 24-h movement guidelines and health in children and adolescents aged 5–17 years: a systematic review and meta-analysis
Source: Front Public Health. 2024 May 7;12:1351972. doi: 10.3389/fpubh.2024.1351972 (PMC11106490; doi:10.3389/fpubh.2024.1351972)
Supplement: Supplementary file 1 [file Data_Sheet_1.docx]

Supplementary Material

**Association between meeting 24-hour movement guidelines and health in children and adolescents aged 5-17 years: A systematic review and meta-analysis**

**HanHua Zhao^1^, Na Wu^2^, Eero A. Haapala^3,4^, Ying Gao^1^***

*** Correspondence:** yigao@zju.edu.cn

**1 List of supplementary materials**

Supplementary Figure 1 The flow Chart of Search Results

Supplementary Figure 2 Forest plot of overall adherence to all three 24-hour movement guidelines and obesity.

Supplementary Table 1 Search strategy

Supplementary Table 2 Cross-sectional associations between adherence to 24-hour movement guidelines and health outcomes

Supplementary Table 3 Longitudinal associations between adherence to 24-hour movement guidelines and health outcomes

Supplementary Table 4 The compliance with 24-hour movement guidelines (%)


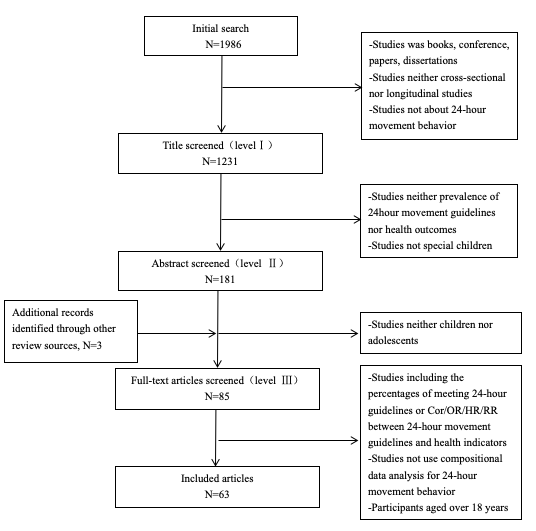


**Supplementary Figure 1** The flow Chart of Search Results


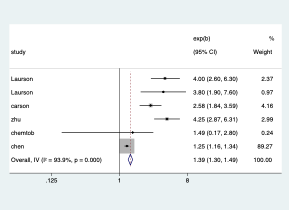


**Supplementary Figure 2** Forest plot of overall adherence to all three 24-hour movement guidelines and obesity.

**Supplementary Table 1** Search strategy

MEDLINE (ovid)

|  | Search strategy | 13 November 2023 |
| --- | --- | --- |
| #1 | ((24?h hour* or 24?h) adj3 (movement or behavio?r or guidelin* or recommendation or period)).kf,tw. | 700 |
| #2 | (“physical activit*” or “active”) kf,tw. | 1290691 |
| #3 | exp Exercise/ | 250155 |
| #4 | exp Sports/ | 218705 |
| #5 | #2 or #3 or #4 | 1562333 |
| #6 | (“sedentary behavior*” or “sedentary” or “sit*” or “screen*” or “watching TV” or “TV watching” or “television*” or “computer use” or “inactivit*”) or (lack adj3 activity) or (low adj3 energy expend*). kf,tw. | 3322076 |
| #7 | (“sleep*” OR “bed time*”) kf,tw. | 239249 |
| #8 | #5 and #6 and #7 | 5229 |
| #9 | #8 or #1 | 5926 |
| #10 | (“teenage*” OR “child*” OR “youth” OR “adolescen*” OR “student*” OR “*school” OR “school age*” OR “childhood”) kf,tw. | 2374431 |
| #11 | #9 and #10 | 2012 |
| #12 | limit #11 to humans | 1665 |
| #13 | limit #12 to English language | 1596 |
| #14 | limit#13 to 2016-2023 | 1264 |

**Supplementary Table 2** Cross-sectional associations between adherence to 24-hour movement guidelines and health outcomes

| Health outcome | Author(year) | General combination of 24-hour movement guidelines and health | Specific combination of 24-hour movement guidelines and health |
| --- | --- | --- | --- |
| **adiposity** | | | |
| body mass index (BMI) | Laurson (2016) | meeting none guidelines (vs all 3) was associated with higher BMI≥95^th^ | meeting the PA were not at increased odds of BMI≥95^th^, regardless of SLEEP or ST |
| body mass index z-score (BMIz) | Roman-vainas (2016) | meeting all 3 or 1-2 guidelines (vs 0) had a lower odds ratio for BMIz | meeting the PA either independently or combined was associated with lower BMIz |
| body mass index z-score | Carson (2017) | meeting none or 1-2 guidelines (vs all 3) was associated with a higher BMIz | meeting either independently or combined was not associated with BMIz |
| body mass index | Jassen (2017) | meeting all 3 guidelines (vs 0) had a lower odds ratio for BMI | for BMI, there was no significant difference either across independently or combined of 24-hour movement guidelines |
| body mass index z-score | Katzmaryzk (2017) | meeting all 3 (vs 0) was associated with lower BMI | meeting the SLEEP was significantly associated with lower odds of obesity |
| body mass index z-score | Chemtob (2021) | meeting none (vs all 3) was cross-sectionally associated with higher BMIz among childhood aged 8-10 years, but not associated with BMIz among early adolescence aged 10-12 years  meeting none (vs all 3) was longitudinally associated with higher BMIz in 2-year changes, but not longitudinally associated with BMIz in 5-year changes among childhood aged 8-10 years | / |
| body mass index z-score | Shi (2020) | meeting all 3 (vs not meeting) had no associations with BMIz | meeting the PA or PA&SLEEP was associated with a healthier body weight in boys |
| body mass index z-score | Guimaraes (2020) | meeting all 3 (vs 0) was not associated with BMIz | meeting the PA&SLEEP (vs not meeting) was associates with a healthier BMIz |
| overweight/obesity  (OW/OB) | Tanaka (2020) | meeting all 3 (vs 0) had lower odds ratio for OW/OB | meeting the ST or combinations including ST were associated with OW/OB |
| overweight/obesity | Chen (2021) | meeting none (vs all 3) had higher odds ratio of being OW/OB for boys in the 4th-6th grades but not for girls, and similarly, for girls in the 7th-9th grades but not for boys  meeting none (vs all 3) had no association with OW/OB in the 10th-12th grades | in 4th-6ths grade, boys meeting the ST or SLEEP and girls meeting the SLEEP or PA&SLEEP had significantly higher odds ratio for OW/OB  meeting either independently or combined were not associated with BMI in 7th-9th and 10th-12th grades |
| overweight/obesity | Yang (2022) | meeting none (vs all 3) had a significant association with higher risk of OW/OB | meeting the SLEEP (vs all 3) was significantly associated with a higher risk of underweight, while meeting the PA or ST was significantly associated with a higher risk of OW/OB |
| body mass index z-score | Jakubec (2020) | meeting all 3 (vs 0) was not associated with BMIz in children and adolescents | meeting either independently or combined were not associated with BMIz in children, while meeting the ST or ST&SLEEP (vs not meeting) was linked to lower BMIz in adolescents |
| obesity | Zhu (2020) | meeting none (vs all 3) was associated with the greatest likelihood of overweight and obesity | meeting the PA, either alone or in combination with ST or SLEEP (vs all 3) was associated with the lowest odds ratio for overweight and obesity |
| body mass index | Garcia (2023) | / | meeting either independently or combined were not associated with BMI |
| weight status | Zhou (2022) | meeting all 3 (vs 0/1) was associated with a lower risk of weight status | meeting the PA, ST, PA&ST, PA&SLEEP was associated with a lower risk of weight status |
| body fat | Katzmaryzk (2017) | meeting all 3 (vs 0) was associated with lower total body fat | meeting specific combination was not significantly associated with total body fat |
| body fat percentage (BF%) | Chemtob (2021) | meeting none (vs all 3) showed cross-sectionally no association with BF% among childhood aged 8-10 years and early adolescence aged 10-12 years  meeting none (vs all 3) was longitudinally associated with higher BF% in 2-year changes but not in 5-year changes among children aged 8-10 years | / |
| body fat percentage (BF%) | Hui (2020) | negative association between number of the guidelines being met and BF% | meeting the PA or PA&SLEEP (vs 0) had negative association with BF% |
| body fat percentage (BF%) | Zhou (2022) | meeting all 3 (vs 0/1) was associated with a lower risk of BF% | meeting the PA, PA&ST, PA&SLEEP was associated with a lower risk of BF% |
| fat mass percentage (FM%) | Jakubec (2020) | meeting all 3 (vs 0) was not associated with FM% | meeting either independently or combined showed no association with FM% in children, while meeting the ST was linked to lower FM% in adolescent |
| fat free mass (FFM) | Zhou (2022) | meeting all 3 (vs not meeting) showed no associations with FFM | meeting either independently or combined was not associated with FFM |
| skeletal muscle mass (SMM) | Zhou (2022) | meeting all 3 (vs not meeting) showed no associations with SMM | meeting either independently or combined was not associated with SMM |
| waist circumference (WC) | Carson (2017) | meeting none (vs all 3) was associated with a higher WC | not meeting the PA or SLEEP&ST was significantly associated with an increased WC |
| waist circumference (WC) | Katzmaryzk (2017) | meeting all 3 (vs 0) was associated with lower WC | meeting specific combination was not significantly associated with WC |
| waist circumference (WC) | Chemtob (2021) | meeting none (vs all 3) showed cross-sectionally no association with WC among childhood aged 8-10 years and early adolescence aged 10-12 years  meeting none (vs all 3) was longitudinally associated with higher WC in 2-year changes but not in 5-year change among childhood aged 8-10 years | / |
| waist circumference (WC) | Leppanen (2021) | meeting all 3 (vs 0) was inversely associated with WC | meeting all 3, PA&ST, PA&SLEEP, PA were inversely associated with WC |
| waist circumference (WC) | Garcia (2023) | / | meeting the ST, PA&ST or ALL 3 were inversely associated with WC |
| waist-to-height ratio (WHtR) | Chemtob (2021) | meeting none (vs all 3) was cross-sectionally associated with higher WHtR among childhood aged 8-10 years, but no association among early adolescence aged 10-12 years  Meeting none (vs all 3) was longitudinally associated with higher WHtR in 2-year changes and in 5-year changed among childhood aged 8-10 years | / |
| **Cardiometabolic health** | | | |
| blood pressure | Carson (2017) | meeting none (vs all 3) was associated with higher systolic blood pressure, but no association with diastolic blood pressure | not meeting the SLEEP&PA (vs meeting) was significantly associated with higher systolic blood pressure and diastolic blood pressure |
| blood pressure | Katzmaryzk (2017) | meeting general combination was not associated with blood pressure | meeting specific combination was not significantly associated with blood pressure |
| blood pressure | Leppanen (2021) | meeting all 3 was associated with diastolic blood pressure, but no association with systolic blood pressure | meeting all 3, PA were inversely associated with blood pressure |
| subcutaneous adipose tissue | Katzmaryzk (2017) | meeting all 3 (vs 0) was associated with lower subcutaneous adipose tissue | meeting specific combination was not significantly associated with subcutaneous adipose tissue |
| visceral adipose tissue | Katzmaryzk (2017) | meeting all 3 (vs 0) was associated with lower visceral adipose tissue | meeting specific combination was not significantly associated with visceral adipose tissue |
| visceral adipose tissue | Jakubec (2020) | meeting all 3 (vs 0) showed no association with visceral adipose tissue | meeting either independently or combined showed no association with visceral adipose tissue in children, while meeting the ST guidelines was linked to lower visceral adipose tissue in adolescent |
| triglycerides | Carson (2017) | meeting none (vs all 3) was associated with higher triglycerides | not meeting the ST&PA (vs meeting) was significantly associated with higher triglycerides |
| triglycerides | Katzmaryzk (2017) | meeting all 3 (vs 0) was associated with lower triglycerides | meeting specific combination showed no association with triglycerides |
| triglycerides | Leppanen (2021) | meeting all 3 (vs 0) was associated with lower triglycerides | meeting specific combination showed no association with triglycerides |
| HDL cholesterol | Carson (2017) | meeting none (vs all 3) was associated with lower HDL cholesterol | not meeting the SLEEP (vs meeting) was associated with higher HDL cholesterol |
| HDL cholesterol | Katzmaryzk (2017) | meeting all 3 (vs 0) was not associated with HDL cholesterol | meeting specific combination showed no association with HDL cholesterol |
| HDL cholesterol | Leppanen (2021) | meeting all 3 (vs 2) showed association with HDL cholesterol | meeting all 3, PA were directly with HDL cholesterol |
| C-reactive protein | Carson (2017) | meeting none (vs all 3) showed association with C-reactive protein | not meeting the SLEEP&PA (vs meeting) was associated with higher C-reactive protein, while not meeting SLEEP was associated with lower C-reactive protein |
| insulin | Carson (2017) | meeting none (vs all 3) was associated with higher insulin | meeting either independently or combined showed no association with insulin |
| insulin | Leppanen (2021) | meeting all 3 (vs 0) was inversely associated with insulin | meeting all 3, PA&ST, PA&SLEEP, PA were inversely associated with insulin |
| glucose | Katzmaryzk (2017) | meeting all 3 (vs 0) was associated with lower glucose | meeting specific combination showed no association with glucose |
| glucose | Leppanen (2021) | meeting all 3 (vs 0) showed no association with glucose | meeting specific combination showed no association with glucose |
| cardiometabolic risk score | Leppanen (2021) | meeting all 3 (vs 0) was inversely associated with cardiometabolic risk score | meeting all 3, PA&ST, PA&SLEEP, PA were inversely associated with cardiometabolic risk score |
| cardiometabolic health markers | Guedes (2022) | meeting none (vs all 3) were twice as likely to have cardiometabolic health risk | meeting the ST, SLEEP, ST&SLEEP (vs all 3) were shown to be significantly more exposed to cardiometabolic health risks |
| **Physical Fitness** | | | |
| aerobic fitness | Carson (2017) | meeting none (vs all 3) was associated with lower aerobic fitness | not meeting the SLEEP (vs meeting) was associated with lower aerobic fitness, while not meeting the SLEEP&ST was associated with higher aerobic fitness. |
| grip strength | Tanaka (2020) | meeting all 3 (vs not meeting) was unrelated to grip strength | meeting either independently or combined showed no association with grip strength |
| handgrip strength | Tapia-serrano | / | meeting all 3 (vs not meeting) was not associated with handgrip strength |
| sit-up | Tanaka (2020) | meeting all 3 (vs not meeting) showed no association with sit-up | meeting the PA or PA&SLEEP was associated with higher sit-up |
| sit-and reach | Tanaka (2020) | meeting all 3(vs not meeting) showed no association with sit-and reach | meeting either independently or combined showed no association with sit-and reach |
| 20-m shuttle run | Tanaka (2020) | meeting all 3 (vs not meeting) showed no association with 20-m shuttle run | meeting the PA was associated with higher 20-m shuttle run |
| general physical fitness | Chen (2022) | meeting all 3 guidelines was associated with higher levels of general physical fitness | meeting the PA was significantly associated with general physical fitness, but not for neither ST nor SLEEP were |
| physical fitness | Tapia-serrano (2022) | / | meeting all 3 (vs not meeting) was more likely to have high/very high for physical fitness |
| cardiorespiratory fitness | Chen (2022) | meeting all 3 was associated with higher levels of cardiorespiratory fitness | meeting the PA was significantly associated with cardiorespiratory fitness, but not for neither ST nor SLEEP were |
| cardiorespiratory fitness | Tapia-serrano (2022) | / | meeting all 3 guidelines (vs not meeting) was more likely to have high/very high for cardiorespiratory fitness |
| muscular strength | Chen (2022) | meeting all 3 was associated with higher levels of muscular strength | meeting the PA was significantly associated with muscular strength, but not for neither ST nor SLEEP were |
| muscular fitness | Tapia-serrano (2022) | / | meeting all 3 (vs not meeting) was more likely to have high/very high for muscular fitness |
| speed and agility | Chen (2022) | meeting all 3 was associated with higher levels of speed and agility | meeting the PA was significantly associated with speed and agility, but not for neither ST nor SLEEP were |
| speed and agility | Tapia-serrano (2022) | / | meeting all 3 (vs not meeting) showed no association with speed and agility |
| flexibility | Chen (2022) | meeting all 3 guidelines showed no association with flexibility | meeting either independently or combined showed no association with flexibility |
| standing long jump | Tapia-serrano (2022) | / | meeting all 3 (vs not meeting) was more likely to have high/very high for standing long jump |
| physical fitness | Cai (2023) | the number of meeting guidelines exhibited a typical dose–response relationship with high level physical fitness index | meeting all 3 (vs 0) with higher-level physical fitness level  meeting PA&ST or PA were better associated with high-level physical fitness index |
| physical health | Hugues (2022) | meeting all 3 guidelines (vs 0) was associated with positive self-reported physical health | different intermediate combinations of the guidelines were all associated with positive self-reported physical health |
| **Mental and social health** | | | |
| behavioral strengths and difficulties (SD) | Carson (2017) | meeting none (vs all 3) was associated with higher SD | not meeting the PA or ST&PA (vs not meeting) was associated with lower SD, while not meeting the SLEEP&ST was associated with higher SD |
| strengths and difficulties questionnaire (SDQ) | Bang (2020) | meeting all 3 was associated with higher odds of positive psychosocial health among youth | meeting the PA or PA&SLEEP (vs not meeting) was associated with higher SDQ |
| emotional problems | Janssen (2017) | meeting all 3 (vs 0) had lower emotional problems | emotional problem scores were lower in the ST group than in  the SLEEP group |
| prosocial behavior | Janssen (2017) | meeting all 3 (vs 0) had higher prosocial behavior | prosocial behavior scores were higher in the PA group than in the ST group |
| life satisfaction | Janssen (2017) | meeting all 3 (vs 0) had higher life satisfaction | for life satisfaction, there was no significant difference either across independently or combined of 24-hour movement guidelines |
| happy | Lee (2018) | meeting all 3 (vs 0) was associated with higher odds of being happy | meeting the PA, ST, PA&ST, PA&SLEEP (vs not meeting) was associated with higher odds of being happy |
| stress | Lee (2018) | meeting all 3 (vs 0) was associated with lower odds of not feeling stress | meeting the PA, ST, PA&ST, PA&SLEEP, ST&SLEEP (vs not meeting) was associated with lower odds of not feeling stress |
| not stressed | Bang (2020) | meeting two or more of the recommendations was associated with lower odds of not stressed | meeting the SLEEP (vs not meeting) was associated with lower odds of not stressed |
| impulsivity | Guerrero (2019) | / | meeting the ST&SLEEP (vs 0) was associated with less impulsive behaviors |
| anxiety | Zhu (2019) | meeting none (vs all 3) had higher odds for anxiety among adolescents aged 12-17 years, but no association among children aged 6-11 years | meeting the PA&ST had higher odds for anxiety among children aged 6-11 years, but no association among adolescent aged 12-17 years |
| depression | Zhu (2019) | meeting none (vs all 3) had higher odds for depression among adolescents aged 12-17 years, but no association among children aged 6-11 years | meeting the ST, ST&SLEEP, PA&SLEEP (vs all 3) had lower odds for depression among children aged 6-11 years, but meeting either special or combined guideline had higher odds for depression among adolescent aged 12-17 years |
| internalizing behaviors | Sampasa (2021) | meeting all 3 (vs 0) was associated with lower risk of internalizing behaviors in youth | meeting the SLEEP or ST&SLEEP (vs 0) was associated with lower risk of internalizing behaviors |
| externalizing behaviors | Sampasa (2021) | meeting all 3 (vs 0) was associated with lower risk of externalizing behaviors in youth | meeting the ST, SLEEP, ST&SLEEP was associated with lower risk of externalizing problems |
| loneliness and sadness | Burns (2020) | meeting all 3 (vs 0) was associated with lower odds of loneliness and sadness | meeting the ST&SLEEP or PA&SLEEP (vs 0) was associated with lower perceived loneliness, and meeting the ST&SLEEP or PA&ST was associated with lower prolonged sadness |
| perceived self-efficacy | Guimaraes (2020) | meeting all 3 (vs 0) was associated with better perceived self-efficacy | meeting the ST, SLEEP, ST &SLEEP (vs not meeting) was associated with better perceived self-efficacy |
| suicidal ideation and suicide attempts | Sampasa (2020) | meeting all 3 (vs 0) was not associated suicidal ideation and suicide attempts in boys aged 11-14 years, nor in girls in both age groups  meeting all 3 (vs 0) was associated with lower odds of suicidal ideation and suicide attempts in boys aged 15-20 years | for boys, there was no association between either independently or combined with suicidal ideation and suicide attempts among 11-14 years and suicidal ideation among 11-14 years, as well as, meeting the PA or SLEEP was associated with lower suicide attempts among 15-20 years  for girls, meeting the ST or ST&PA was associated with suicidal ideation among 11-14 years and meeting the ST&SLEEP was associated with suicidal ideation among 15-20 years  meeting ST was associated with lower suicide attempts among 11-14 years, meeting PA was associated with lower suicide attempts among 15-20 years |
| depressive symptoms | Lu (2021) | the greater number of meeting recommendation is linked to a lower likelihood of depressive symptoms | meeting the SLEEP&ST is linked to the lowest odds of developing depressive symptoms |
| anxiety | Lu (2021) | the greater number of meeting recommendation is linked to a lower likelihood of depressive anxiety | Meeting the SLEEP&ST is linked to the lowest odds of developing of anxiety |
| subjective wellbeing | Sun (2023) | meeting all 3 (vs 0) was associated with better subjective wellbeing | meeting the PA, ST, SLEEP was associated with better greater subjective wellbeing |
| good mental health | Bang (2020) | meeting two or more of the recommendations was not associated with mental health | meeting the SLEEP (vs not meeting) had higher odds of good mental health |
| psychosocial health | Fung (2022) | / | meeting the PA evidenced greater levels of externalizing problems |
| mental health | Hugues (2022) | meeting all 3 guidelines (vs 0) was associated with positive self-reported mental health | different intermediate combinations of the guidelines were all associated with positive self-reported mental health, except for ST |
| mental health problems | Zhang (2023) | the severity of anxiety and depressive symptoms significantly decreased as the number of recommendations met increased for all 3 (vs 0) | / |
| substance use | Sampasa (2021) | meeting all 3 (vs 0) showed no association with substance uses (cigarette smoking, alcohol consumption, cannabis use), only less cannabis use was | meeting the PA or PA&SLEEP was associated with lower odds of cigarette smoking, and meeting the PA or PA&ST was associated with lower odds of cannabis use, as well as meeting the ST, SLEEP, PA&ST, ST&SLEEP was associated with lower odds of alcohol consumption  meeting the SLEEP or ST&SLEEP was associated with lower odds of cannabis among 11-14 years, either independently or combined was not associated with cannabis among 15-20 years |
| internet Addiction | Ma (2022) | meeting 0 (vs all 3) was associated with higher risk of Internet Addiction | meeting either independently or combined (vs all 3) was not associated with higher risk of internet addiction |
| **Health related quality of life** | | | |
| health related quality of life (HRQoL) | Sampasa (2017) | / | meeting the ST, ST&SLEEP, all 3 (vs not meeting) had better HRQoL |
| health related quality of life (HRQoL) | Khan (2021) | meeting all 3 (vs 0) was associated with higher overall HRQoL score as well as physical and psychosocial scores | meeting the SLEEP&PA, ST&SLEEP was associated with higher scores for HRQoL |
| health related quality of life (HRQoL) | Guimaraes (2020) | meeting all 3 (vs 0) was associated with better HRQoL | meeting the PA, SLEEP, PA&ST, PA&SLEEP (vs not meeting) was associated with better HRQoL |
| **Perceived health** | | | |
| perceived health | Guimaraes (2020) | meeting all 3 (vs 0) showed no association with perceived health | meeting the PA or PA&SLEEP (vs not meeting) was associated with better perceived health |
| Self-rated health | Kyan（2022） | / | among elementary school students，meeting the ST&SLEEP was associated with a high prevalence of good health  Among junior high school, meeting the PA. SLEEP, ST&SLEEP, PA&SLEEP and ALL 3 were associated with a high prevalence of good health |
| **Academic achievement** | | | |
| academic achievement | Watson (2022) | meeting all 3 (vs 0) had a stronger positive association with academic achievement (literacy and numeracy) | meeting the PA was important for numeracy achievement, as well as, meeting the ST&SLEEP had the strongest positive association with literacy achievement |
| academic performance | Howie (2020) | meeting at least 2 out of the 3 guidelines was associated with better academic performance. | meeting the ST was associated with higher average academic index and English scores, and higher time in SB was associated with poorer academic performance |
| academic achievement | Sapmapsa (2022) | meeting all 3 had higher academic achievement | meeting the PA and SLEEP guidelines, both independently or together, was associated with higher academic achievement |
| academic performance | Lien (2019) | / | middle school student who met all 3, ST, SLEEP (vs 0) displayed better academic performance  high school students who met ST, SLEEP (vs 0) displayed better academic performance |
| **Cognitive development** | | | |
| global cognition | Walsh (2018) | meeting all 3 (vs 0) was significantly positive association with global cognition | significant positive association between global cognition and meeting the ST&SLEEP or SLEEP |
| cognitive development | Fung (2022) | / | meeting ST, SLEEP&ST, or all 3 (vs not meeting) evidenced superior fluid intelligence, crystallized intelligence, and composite cognition scores |
| cognitive difficulties | Lu (2023) | / | meeting all 3 guidelines was most strongly associated with having no cognitive difficulties  meeting the PA&SLEEP was associated with greater odds of being classified as having no cognitive difficulties |
| executive function | Zeng (2022) | the number of guidelines met positive related to completed categories, shifting efficiency, negatively related to non-perseverative errors | not meeting the PA or SLEEP had lower scores in categories and shifting efficiency  meeting the ST had no significant association with any processes of executive function |
| gray matter volumes | Fung (2023) | / | meeting the ST&SLEEP (vs not meeting) consistently showed greater total cortical volumes |
| **Dietary patterns** | | | |
| dietary patterns | Thivel (2019) | / | meeting the ST guidelines is strongly associated with desirable dietary patterns |
| **myopia** | | | |
| myopia | Zhao (2023) | the number of meetings 24hour movement guideline was negatively associated with reduced risk of myopia | meeting the SLEEP, ST&SLEEP and all 3 guidelines were associated with significantly decreased risk of myopia |

**Supplementary Table 3** Longitudinal associations between adherence to 24-hour movement guidelines and health outcomes

| Health outcome | Author(year) | General combination of 24-hour movement guidelines and health | Specific combination of 24-hour movement guidelines and health |
| --- | --- | --- | --- |
| **adiposity** | | | |
| body mass index (BMI) | Chemtob (2021) | compared to meeting all 3 in childhood (age 8-10), those meeting 0 had significantly higher BMI on all measures examined in early adolescence (age 10-12 years)  compared to meeting all 3 in childhood (age 8-10), those meeting 1 had higher BMI on all measures examined in adolescence (age 15-17 years) | / |
| body mass index z-score (zBMI) | Fung (2023) | / | meeting the PA&SLEEP at baseline was inversely associated with zBMI at 2 years follow up |
| body fat percentage (BF%) | Chemtob (2021) | compared to meeting all 3 in childhood (age 8-10), those meeting 0 had significantly higher BF% on all measures examined in early adolescence (age 10-12 years)  compared to meeting all 3 in childhood (age 8-10), those meeting 1 had higher BF% on all measures examined in adolescence (age 15-17 years) | / |
| waist circumference | Chemtob (2021) | compared to meeting all 3 in childhood (age 8-10), those meeting 0 had significantly higher waist circumference on all measures examined in early adolescence (age 10-12 years)  compared to meeting all 3 in childhood (age 8-10), those meeting 1 had higher waist circumference on all measures examined in adolescence (age 15-17 years) | / |
| waist circumference | Leppanen(2021) | meeting ALL 3(vs 0) at baseline was inversely associated with waist circumference at 2 years follow-up |  |
| waist-to-height ratio (WHtR) | Chemtob (2021) | compared to meeting all 3 in childhood (age 8-10), those meeting 0 had significantly higher WHtR on all measures examined in early adolescence (age 10-12 years)  Compared to meeting all 3 in childhood (age 8-10), those meeting 1 had higher WHtR on all measures examined in adolescence (age 15-17 years) | / |
| **Cardiometabolic health** | | | |
| cardiometabolic risk score | Leppänen (2021) | meeting all3 (vs 0) at baseline was inversely associated with c at 2 years follow-up | meeting all 3, PA&ST, PA&SLEEP, ST&SLEEP, PA at baseline was inversely associated with cardiometabolic risk at 2 years follow-up |
| insulin | Leppänen (2021) | meeting all 3 (vs 0) at baseline was inversely associated with insulin at 2 years follow-up | meeting all 3, PA&ST, PA&SLEEP, PA at baseline was inversely associated with insulin at 2 years follow-up |
| glucose | Leppänen (2021) | meeting all 3 (vs 0) at baseline showed no association with glucose at 2 years follow-up | meeting all 3, PA&ST, PA at baseline was inversely associated with glucose at 2 years follow-up |
| triglycerides | Leppänen (2021) | meeting all 3 (vs 0) at baseline showed no association with triglycerides at 2 years follow-up | either independently or combined guidelines at baseline showed no association with triglycerides at 2 years follow-up |
| HDL cholesterol | Leppänen (2021) | meeting all 3 (vs 0) at baseline showed no association with HDL cholesterol at 2 years follow-up | either independently or combined guidelines at baseline showed no association with HDL cholesterol at 2 years follow-up |
| systolic blood pressure | Leppänen (2021) | meeting all 3 (vs 0) at baseline showed no association with systolic blood pressure at 2 years follow-up | either independently or combined guidelines at baseline showed no association with systolic blood pressure at 2 years follow-up |
| diastolic blood pressure | Leppänen (2021) | meeting all 3 (vs 0) at baseline showed no association with diastolic blood pressur at 2 years follow-up | either independently or combined guidelines at baseline showed no association with diastolic blood pressur at 2 years follow-up |
| **Mental and social health** | | | |
| cognition | Fung (2023) | / | meeting the PA&SLEEP at baseline was inversely associated with cognition at 2 years follow up |
| psychosocial | Fung (2023) | / | meeting the PA&SLEEP at baseline was inversely associated with psychosocial at 2 years follow up |
| gray matter volumes | Fung (2023) | / | meeting the PA&SLEEP at baseline was inversely associated with gray matter volumes at 2 years follow up |
| anxiety | Zhang (2023) | adolescents who met three recommendations at baseline displayed lower anxiety symptoms six months later than those who did not | / |
| depression | Zhang (2023) | adolescents who met three recommendations at baseline displayed lower depressive symptoms six months later than those who did not | / |

**Supplementary Table 4** The compliance with 24-hour movement guidelines (%)

| **Author and Publication Year** | **Country** | **PA** | **ST** | **SLEEP** | **PA+ST** | **PA+SLEEP** | **ST+SLEEP** | **0** | **1** | **2** | **3** |
| --- | --- | --- | --- | --- | --- | --- | --- | --- | --- | --- | --- |
| Laurson et al.,2015 | America | 30 | 70 | 30 | / | / | / | / | / | / | / |
| Roman-Viñas et al., 2016 | 12 countries | 44.1 | 39.3 | 41.9 | 16.6 | 18.2 | 16.7 | 19 | / | / | 7.2 |
| Carson et al.,2017 | Canada | 30.7 | 25.4 | 64.2 | 2.9 | 11.3 | 22.8 | 11 | 34.9 | 37 | 17.1 |
| Janssen et al.,2017 | Canada | 33.3 | 8 | 65.3 | / | / | / | 21.4 | 51.1 | 24.9 | 2.6 |
| Katzmarzyk et al.,2017 | America | 30.8 | 35 | 52.4 | 14 | 18.2 | 21.3 | 26.9 | 36.4 | 28.3 | 8.4 |
| Roberts et al.,2017 | Canada | 36 | 49.3 | 75.3 | 20.7 | 28.1 | 40 | 10.7 | 35.5 | 36.3 | 17.5 |
| Sampasa-Kanyinga et al.,2017 | 12 countries | 44.1 | 39.3 | 41.9 | / | / | / | 18.9 | 38.9 | 35 | 7.2 |
| Lee et al.,2018 | Korea | 5 | 39.4 | 67.6 | 2.4 | 3.3 | 26 | 18.1 | 27 | 53.4 | 1.6 |
| Walsh et al.,2018 | America | 17.5 | 36.6 | 51 | 7.3 | 9.5 | 22.5 | 29.4 | 70.6 | / | 4.8 |
| Guerrero et al.,2019 | Canada | 5.5 | 11.5 | 23.7 | 2.5 | 4.7 | 28.7 | 29.4 | 24.9 | 40.7 | 2.5 |
| Manyanga et al.,2019 | Mozambique | 91.3 | 25.8 | 39.6 | 25.3 | 36.1 | 11.2 | 5 | 43.9 | 40.2 | 10.8 |
| Pearson et al.,2019 | England | 40.5 | 23.1 | 89.3 | 10.5 | 36.3 | 21.6 | / | / | / | 9.7 |
| Thivel et al.,2019 | 12 countries | 44 | 39 | 42 | 17 | 18 | 17 | 19 | / | / | 7 |
| Zhu et al.,2019^*^ | America | 23.0 | 17.8 | 66.2 | 5.3 | 15.9 | 13.4 | 23.2 | 50.6 | 22.0 | 4.2 |
| Zhu et al.,2020 | America | 19.1 | 76.2 | 58.1 | 15.3 | 11.6 | 44.9 | 9.1 | 37.9 | 43.6 | 9.4 |
| Ying et al.,2020 | China | 2.8 | 96.3 | 7.9 | / | / | / | 5.1 | 85.8 | 8.8 | 0.3 |
| Chemtob et al.,2021* | Canada | 22.0 | 31.8 | 93.1 | 8.2 | 20.6 | 31.6 | 5.3 | 50.1 | 36.7 | 7.9 |
| Sampasa-Kanyinga et al.,2021 | Canada | 7.7 | 13.8 | 15.4 | 5.9 | 4.6 | 8.6 | 39 | / | / | 5 |
| Sampasa-Kanyinga et al.,2021 | America | 16.4 | 35.1 | 47.4 | 6.4 | 8.1 | 20.5 | 32.2 | / | / | 3.9 |
| Tanaka et al.,2020 | Japan | 60.9 | 20.2 | 68.3 | 12.3 | 45.7 | 13.6 | 13.2 | / | / | 9.1 |
| Waston et al.,2022* | Australia | 60.6 | 25.6 | 73.1 | 20.9 | 45.8 | 24.4 | / | / | / | 15.5 |
| Toledo-Vargas et al.,2020* | Chile | 9.2 | / | 51.1 | 3.0 | 4.1 | 12.1 | 31.3 | 68.7 | 17.0 | 2.3 |
| Tanaka et al.,2020 | Japan | 60.4 | 21.5 | 68.7 | 13.5 | 44.9 | 16.1 | 13.2 | / | / | 10.5 |
| Buchan et al.,2020 | Canada | 35.9 | 6.4 | 50.8 | / | / | / | / | / | / | 1.28 |
| Chen et al.,2021 | China | 11.8 | 65.3 | 44.4 | 8.4 | 7.1 | 28.5 | / | / | / | 5.1 |
| Bruns et al.,2020 | America | 15.6 | 57.2 | 24.6 | / | / | / | 20.9 | 49.7 | 24.4 | 5 |
| Chen et al.,2020 | China | 9.9 | 65.2 | 37.2 | / | / | / | / | / | / | 2.9 |
| Tapia-Serrano et al.,2021 | Spain | 38 | 15.8 | 81.3 | 6.6 | 31.6 | 12.5 | 10.2 | 49.9 | 34.5 | 5.4 |
| Khan et al.,2021 | Australia | 9.3 | 26 | 74.2 | 3 | 6.7 | 20.2 | 18.1 | 56.8 | 22.7 | 2.4 |
| Shi et al.,2020 | Hong Kong, China | 9.1 | 31.2 | 38.6 | / | / | / | / | / | / | 1 |
| Howie et al.,2020 | Australia | 21 | 11 | 74 | / | / | / | / | / | 23 | 2 |
| Guimarães et al.,2020 | Canada | 12 | 34.4 | 60.5 | / | / | / | 25.4 | 44.6 | 27.9 | 2.2 |
| Sampasa-Kanyinga et al.,2020 | Canada | 22.1 | 35.4 | 48.4 | 10.4 | 10.8 | 17.5 | 27.7 | / | / | 5.2 |
| Rubín et al., | Czech | 29.9 | 35.3 | 27.7 | 11.1 | 10.5 | 10.5 | 34.6 | / | / | 4.4 |
| Friel et al.,2020 | America | 23 | 32.9 | 86 | / | / | / | 8.8 | 49.4 | 33 | 8.8 |
| Tapia-Serrano et al.,2022 | Spain | 38 | 15.7 | 82.7 | 6.7 | 32.2 | 12.9 | 9.6 | 50 | 34.5 | 5.8 |
| Zeng et al.,2022 | China | 15.7 | 82.4 | 72.1 | 14.1 | 11.7 | 60.1 | 5.6 | 28.7 | 55.6 | 10.1 |
| Hui et al.,2020 | eight Asia countries | 12.5 | 6.2 | 19.6 | 2.4 | 4.4 | 2.9 | 51.1 | / | / | 0.8 |
| Cai et al.,2023 | China | 3.8 | 40.4 | 7.1 | 8.8 | 2.4 | 18 | 12.2 | 51.2 | 29.2 | 7.3 |
| Swindell et al.,2022 | Kenya | 66 | 57 | 20 | 38 | 14 | 10 | 12 | / | / | 7 |
| Lu et al.,2021 | China | 2.5 | 41.6 | 8.8 | 4.8 | 1.4 | 15.7 | 21.9 | 52.9 | 21.9 | 3.2 |
| Kyan et al.,2022 | Japan | 3.1 | 4.7 | 42.2 | 1 | 12.2 | 12.3 | 20.7 | / | / | 3.8 |
| Da Costa et al.,2021 | Brazil | 9.6 | 12 | 20.8 | 4.3 | 8.1 | 9.2 | 32.9 | / | / | 3.11 |
| Da Costa et al.,2021 | Brazil | 3.6 | / | 20.5 | / | 1.6 | / | / | / | / | / |
| Lu et al.,2023 | China | 11 | 16.1 | 10.9 | 7 | 3.8 | 4.7 | 43.7 | / | / | 2.9 |
| Zhao et al.,2023 | China | 0.84 | 26.7 | 11.2 | 1.7 | 0.5 | 42.7 | 11.5 | / | / | 4.9 |
| Leppanen et al.,2021 | Finland | 79.2 | 73.2 | 90.4 | 58.3 | 71.7 | 66.1 | / | / | / | 52.5 |
| Sun et al.,2023 | China | 2 | 39.8 | 9.3 | 4.9 | 0.2 | 12.3 | 30 | 51.1 | 17.4 | 1.5 |
| Hansen et al.,2022 | Germany | 37 | 35 | 50 | / | / | / | 25 | / | / | 9.7 |
| Salas et al.,2023 | Spain | 31.9 | 50.5 | 82.4 | / | / | / | 9.1 | 34.7 | 38.6 | 17.6 |
| Lien et al.,2020 | Canada | 23.1 | 33.5 | 33.8 | / | / | / | 39 | / | / | 5.1 |
| Guedes et al.,2022 | Brazil | 21.2 | 22.2 | 34.9 | 8.5 | 13.6 | 12.1 | 9.3 | 65.5 | 20.4 | 4.8 |
| Bang et al.,2020 | Canada | 40.1 | 29.9 | 77.3 | 12.3 | 32.5 | 24.6 | / | / | / | 10.7 |
| Fung et al.,2023 | America | 6 | 12 | 23 | 2 | 5 | 17 | 31 | / | / | 4 |
| Suchert et al.,2023 | Germany | 14 | 22 | 34.2 | / | / | / | / | / | / | 2.3 |
| Sampasa-Kanyinga et al.,2022 | Canada | 20.8 | 30.3 | 33.8 | / | / | / | 39.2 | 37.2 | 18.7 | 4.4 |
| Zhou et al.,2022 | China | 55.4 | 63.4 | 68.4 | 36.9 | 38.2 | 44.2 | 5.9 | / | / | 26.1 |
| Ma et al.,2022 | China | 2.4 | 39.2 | 9.6 | 5.1 | 0.9 | 14.3 | 25.3 | 51.2 | 20.3 | 3.2 |
| Zhang et al.,2023 | China | 33.3 | 68.1 | 64.7 | 23.1 | 21.5 | 46.2 | 9.2 | 31 | 44.3 | 15.5 |
| Garcia et al.,2023 | America | 33.2 | 40.8 | 52.3 | 13.3 | 18.1 | 21 | 18.7 | / | / | 7.4 |
| Jakubec et al.,2020 | Czech | 12.8 | 18.3 | 11.2 | 6.6 | 6.1 | 6.0 | 34.6 | 37 | 18.7 | 4.4 |
| Yang et al.,2022 | China | 35.7 | 17.8 | 36.4 | 5.6 | 13.3 | 6.3 | / | / | / | 2.1 |
|  | Averaged | 28.8 | 37.3 | 52.8 | 10.8 | 17.3 | 20.7 | 19.7 | 46.6 | 30.3 | 7.2 |

*no direct results, indirect calculations M=（M_1_N_1_+M_2_N_2_）/（N_1_+N_2_）
